# Pes planus level affects counter movement jump performance: A study on amateur male and female volleyball players

**DOI:** 10.1097/MD.0000000000038683

**Published:** 2024-06-21

**Authors:** Ahmet Kurtoğlu, Bekir Çar, Özgür Eken, Gözde Demir, Musa Türkmen, Edi Setiawan, Madawi H. Alotaibi, Safaa Mostafa Elkholi

**Affiliations:** a Faculty of Sport Science, Department of Coaching Education, Bandirma Onyedi Eylul University, Balikesir, Turkey; b Faculty of Sport Science, Department of Coaching Education, Bandirma Onyedi Eylul University, Balikesir, Turkey; c Department of Physical Education and Sport Teaching, Inonu University, Malatya, Turkey; d Department of physical education, health and recreation, Faculty of Teacher Training and Education, Universitas Suryakancana, Sukabumi, Indonesia; e Department of Rehabilitation Sciences, College of Health and Rehabilitation Sciences, Princess Nourah bint Abdulrahman University, Riyadh, Saudi Arabia.

**Keywords:** counter movement jump, pes planus, volleyball players

## Abstract

The aim of this study was to investigate the effect of pes planus level on counter movement jump (CMJ) performance parameters in amateur female and male volleyball players. In this context, amateur volleyball players aged between 18 and 23 years actively playing in the university school volleyball team were included in the study. Pes planus levels of the participants were analyzed using the navicular drop test (NDT). My Jump Lab application was used for CMJ measurement. Within the scope of CMJ, the participants’ jump height, force, relative force, power, relative power, average speed, take-off speed, impulse, and flying time were analyzed. According to the linear regression results between NDT and CMJ parameters, force in males (*t* = 12.93, *P* = .049) and average speed in females (*t* = −3.52, *P* = .017) were significantly associated with NDT. NDT was similar in men and women (*P* > .05). However, all CMJ parameters were highly different between genders (*P* < .001). In the correlation analysis between sport age and physical characteristics and CMJ parameters; height (*r* = .386, *P* = .046), weight (*r* = .569, *P* = .002), leg length (*r* = .389, *P* = .045), foot length (*r* =. 558, *P* = .005), foot width (*r* = .478, *P* = .018), force (*r* = .407, *P* = .039), impulse (*r* = .460, *P* = .018) parameters, and sport age. The results suggest that the average speed in females and force in males both significantly influenced NDT, highlighting the significance of both factors in predicting NDT scores. Moreover, all CMJ measures showed significant variations between genders, although the NDT scores did not. Furthermore, the correlation analysis demonstrated a strong correlation between a number of physical attributes and CMJ parameters, highlighting the multifaceted nature of athletic performance and indicating the possible impact of these attributes on CMJ results.

## 1. Introduction

Vertical jump performance is recognized as an important criterion in many sports branches.^[1]^ Especially in basketball, volleyball, football, athletics, and many other sports, the vertical jumping ability of players or athletes is an important factor determining their performance. In these sports, vertical jumping ability is usually associated with basic physical characteristics such as speed,^[2]^ strength,^[3,4]^ explosiveness,^[5]^ and coordination.^[6]^ For example, in fast and athletic sports such as basketball and volleyball, it is critical for players to have a high jumping ability to pass or block opposing players.^[7,8]^ In football, vertical leap performance plays a vital role for players to excel in air ball challenges or to have high power when shooting.^[9]^ In athletics, a strong vertical jumping ability is necessary for success in disciplines such as high jump, pole vault, and triple jump.^[10]^

Vertical jump performance is influenced by a number of fundamental physical characteristics.^[11]^ Among these, muscle strength, speed, explosiveness, flexibility, balance, and coordination play a particularly important role. Muscle strength, especially the strength of the leg and hip muscles, largely determines vertical jumping ability. The strength of these muscle groups enables to produce more force at the beginning and end points of the jumping movement.^[12]^ Speed and explosiveness enable the jumping movement to be performed quickly and powerfully.^[13]^ These characteristics increase the acceleration in the jumping movement, resulting in a higher jump.^[14]^ At the same time, some studies have emphasized that foot morphometry is also important for vertical jump performance.^[15]^ Grozier et al^[16]^ examined the propulsion kinetics of medial longitudinal arch (MLA) height during vertical jumping; it was concluded that vertical jump stiffness was higher in individuals with lower MLA flexibility. And although it was concluded that active and passive structures of the foot, have effects on vertical jump performance, they argued that additional research should be conducted to better understand the contribution of MLA flexibility to jump performance.^[16]^ Zhao et al^[17]^ tested the relationship between arch height and physical performance in adult males and concluded that there was a negative relationship between MLA index and vertical jump performance.^[17]^ Just as arc height affects some performance characteristics, some demographic characteristics also affect arc height. In their study, Zhao et al^[18]^ reported that arch flexibility decreased gradually with increasing age, women had a higher arch height, and body mass index (BMI) was also associated with arch height. The lack of MLA of the foot, which causes the affected foot region to come into closer contact with the ground, is known as pes planus or flat foot^.[19]^

In a recent study, there was a positive correlation between MLA level and BMI in men and women, and as BMI increased, MLA level increased in both genders.^[20]^

When the literature was analyzed, it was found that demographic and physical characteristics affect MLA flexibility and stiffness, whereas MLA flexibility and stiffness affect performance. Many studies have focused on the vertical jump height of MLA flexibility,^[14,16,18]^ but the number of studies examining the effect of MLA flexibility on parameters such as force generated during vertical jump performance, hover time, power, relative power, and take-off speed was found to be limited. Therefore, the aim of this study was to investigate the effect of navicular flexibility on the parameters affecting counter movement jump (CMJ) performance. In this context, the hypotheses of our research are:

H_1a_: “CMJ parameters are affected by navicular drop test (NDT) results.”

H_1b_: “Time spent in volleyball (sport age) indirectly affects vertical jump performance by enabling some physical characteristics to be shaped differently.”

## 2. Methods

### 2.1. Participants

In this study, an experimental method was used among quantitative data collection techniques. In this context, the participants who were actively competing in the Bandirma Onyedi Eylul University Volleyball team were included in the study. At the Table 1 shows the demographic characteristics of the participants; accordingly, the mean age of male participants (n = 12) was 20.66 ± 1.49 years, mean height was 179.08 ± 6.99 cm, mean body weight was 75.66 ± 10. 29 kg, BMI mean 23.53 ± 2.35 kg/m^2^, leg length 100.33 ± 4.33 cm, leg length at 90° flexion 70.79 ± 3.29 cm, foot length 26.27 ± 1.05 cm, foot width 80.45 ± 4.87 cm, and sport age 9.50 ± 2.71 years. The mean age of female participants (n = 15) was 19.86 ± 1.59 years, mean height was 169.26 ± 7.75 cm, mean body weight was 63.33 ± 6.32 kg, mean BMI was 22.20 ± 2.96 kg/m^2^, leg length was 97.80 ± 4.62 cm, leg length at 90° flexion was 66.83 ± 4.64 cm, foot length was 24.03 ± 1.46 cm, foot width was 68.07 ± 7.12 cm, and sport age was 7.86 ± 1.80 years. Participants who were licensed competitors in volleyball for at least 3 years were included in the study. Participants with any problem in navicular bone, subtalar joint deformity, any problem in the metatarsal bones, ankle, knee joint, and hip joint problems, anterior cruciate ligament, meniscus, and lateral–medial collateral ligament injuries in the last 6 months and treated for this reason, and current flu infection were excluded from the study. The minimum sample size was determined using G-Power software (version 3.1.9.7; Kiel, Germany).^[21]^ Accordingly, *F* tests in the G-Power programme: linear multiple regression: Fixed model, *R*^2^ deviation from zero (a priori: compute required sample size-given α, power, and effect size). When the effect size (*f*^2^) = 1.15, α err prob = 0.05, power (1-β err prob) = 0.8, number of prediction = 9, it was determined that a total of 24 participants should participate in the study (81.6% actual power).

**Table 1:** Demographic information of participants.

Parameters	Male	Female
	n = 12	n = 15
	Mean ± SD	Mean ± SD
Age (yr)	20.66 ± 1.49	19.86 ± 1.59
Height (cm)	179.08 ± 6.99	169.26 ± 7.75
Weight (kg)	75.66 ± 10.29	63.33 ± 6.32
BMI (kg/m^2^)	23.53 ± 2.35	22.20^*^ ± 2.96
Leg length (cm)	100.33 ± 4.33	97.80 ± 4.62
Leg length at 90° flexion (cm)	70.79 ± 3.29	66.83 ± 4.64
Foot length (cm)	26.27 ± 1.05	24.03 ± 1.46
Foot width (cm)	80.45 ± 4.87	68.07 ± 7.12
Sport age (yr)	9.50 ± 2.71	7.86 ± 1.80

BMI = body mass index.

*P < 0.05.

Necessary explanations about the purpose, reason, and hypotheses of the study were made by the responsible researcher to all participants who would participate in the study. Informed consent forms were signed by all participants. Within the scope of this research, the necessary permissions were obtained from the Inonu University Health Sciences Non-Interventional Clinical Research Ethics Committee with decision number 5156 (date of decision: November 28, 2023). In addition, the present study was conducted in accordance with the principles set out in the Declaration of Helsinki.^[22]^

### 2.2. Experimental design of study

**Figure 1.**
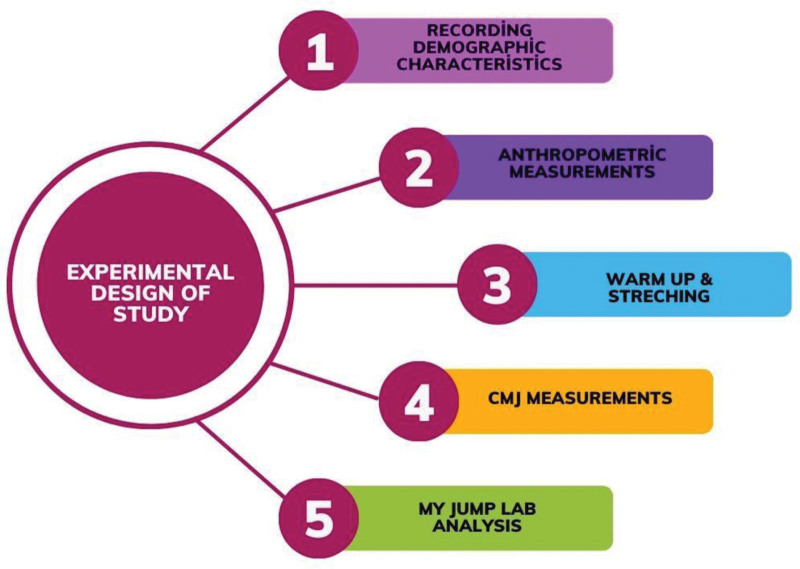
Experimental design of study.

For the present study, demographic characteristics, leg length at full extension and leg length at 90° flexion of the knee joint were recorded. The NDT was performed to determine the level of pes planus. The test protocol started with a 10-minute warm-up that comprised dynamic stretching, submaximal vertical jumps, and jogging. This was based on comparable jumping warm-up protocols from earlier investigations.^[23,24]^ The warm-up consisted of 5 minutes of running, followed by 6 submaximal vertical jumps (free arms, with arm swing and reach) and dynamic stretches (quadriceps, hamstrings, gluteal muscles, calves, shoulders, and back) with movement through their range of motion (not held). After this process, 2 vertical jump performances were recorded. A passive rest period of 2 minutes was provided between jumps. My Jump Lab application was used for the vertical jump performances. Before the study, the participants were warned not to consume any food or drink, except water, for at least 3 hours before the tests. They were asked to avoid activities that could cause fatigue prior to the tests. All tests were performed in an indoor sports hall between 17:00 and 18:00 before routine volleyball training. Male and female athletes were tested on separate days so that the waiting time would not negatively affect the performance results (Fig. 1).

### 2.3. Data collection tools

#### 2.3.1. CMJ performance

The participants’ vertical jump performance was determined using the CMJ. CMJ was performed using My Jump Lab, a smartphone application. In the reliability and validity study of My Jump by Yingling et al, it was found that the consistency between jump height (ICC = 0.813; 95% CI [0.747–0.863]) and peak power (ICC = 0.926; 95% CI [0.897–0.947]) according to CMJ performance with Vertec and My Jump applications was moderate to near perfect. In conclusion, it was concluded that My Jump provides similar results to expensive laboratory instruments and that the test results are reliable.^[25]^ Accordingly, 240 Hz videos of the participants were recorded with the help of Ipad. In the recorded video, the take-off and landing frames of the participants were identified and defined in the literature.

$$\begin{array}{l} h=t^{2}\times 1.22625 \end{array}$$ (1)

**Figure 2.**
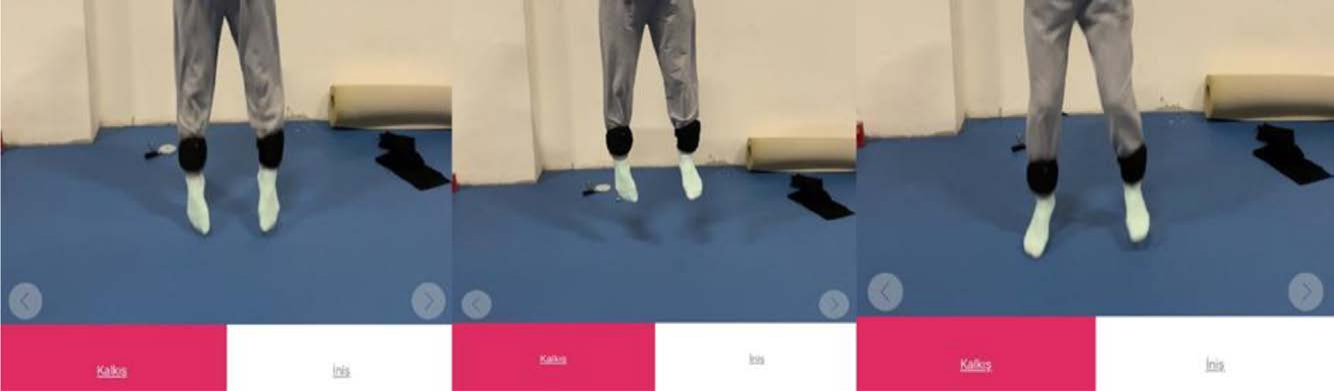
CMJ performance measurement with My Jump 2 application. CMJ = counter movement jump.

The flight time of the CMJ was calculated by converting this to jump height using the equation (where h is the jump height in meters and t is the flight time of the jump in seconds).^[26]^ The participant was instructed to begin the CMJ performance in an upright position, execute a forward fall action with knee and hip flexion, jump vertically upward, and then swiftly and violently execute knee and hip extension to land.^[27]^ The Ipad was fixed to a tripod 1.5 m away from the sample. To determine jump height and flight time, once a jump was recorded, the first frame in which both feet were off the ground (take-off phase) and the first frame in which at least 1 foot touched the ground (landing phase) were chosen in My Jump. The study employed an iPhone 6s with a 240 Hz high-speed camera with 720 p resolution. Each participant was asked to perform 2 repetitions and the best value was recorded (Fig. 2).

#### 2.3.2. Navicular drop test (NDT)

First, the participants were seated barefoot and the most noticeable region of the navicular tubercle was marked with a fine-tipped pen. After that, the subjects were instructed to stand up while maintaining their subtalar neutral posture. While standing, the distance (in millimeters) between the navicular tubercle and the floor was measured. “Navicular drop” was the distance that was reported.^[28]^

### 2.4. Statistical analysis

In the current study, normality distribution of the data was performed using Q-Q plot graphs with the Shapiro–Wilk test. The relationship between NDT and CMJ parameters (jump height, force, relative force, power, relative power, average speed, take-off speed, impulse, and flying time) was determined by linear regression analysis. The results of the linear regression analysis were presented as estimates and standard errors. The *R*^2^ results were also provided for the effect size. An Independent sample *t* test was used to analyze the differences of NDT and CMJ parameters according to gender. The results are presented as mean and standard deviation. The effect size was calculated according to Cohen d formula. The effect size (ES) magnitude was defined as follows: <0.2 = trivial, 0.2 to 0.6 = small effect, >0.6 to 1.2 = moderate effect, >1.2 to 2.0 = large effect, and >2.0 = very large.^[29]^ GraphPad Prism 8 (San Diego, CA, USA) was used for graphical representations of the tests in the study. Interpolation of unknowns from the standard curve and 95% confidence intervals of the line of best fit were obtained when creating graphs for the linear regression analysis. Statistical analyses were performed with RStudio (Version 2023.12.1 + 40, PBS, Boston, MA) and SPSS (version 26, IBM, New York, NY). The significance level was set as 0.05.

## 3. Results

Table 2 shows the results of the correlation analyses between the participants’ sports age, physical characteristics and CMJ parameters. Accordingly, height (*r* = .386, *P* = .046), weight (*r* = .569, *P* = .002), leg length (*r* = .389, *P* = .045), foot length (*r* = .558, *P* = .005), foot width (*r* = .478, *P = *.018), force (*r* = .407, *P* = .039), and impulse (*r* = .460, *P* = .018) values increased significantly as sport age increased.

**Table 2:** Correlation between sport age and physical characteristics and CMJ parameters.

Parameters	Sport age
Height (cm)	*r* = .386, *P* = .046^*^
Weight (kg)	*r* = .569, *P* = .002^**^
Leg length (cm)	*r* = .389, *P* = .045^*^
Leg length at 90° flexion (cm)	*r* = .355, *P* = .069
Foot length (cm)	*r* = .558, *P* = .005^**^
Foot width (cm)	*r* = .478, *P* = .018^*^
NDT (mm)	*r* = −.301, *P* = .128
Jump height (cm)	*r* = .239, *P* = .240
Force (N)	*r* = .407, *P* = .039^*^
Relative force (N/kg)	*r* = .224, *P* = .272
Power (W)	*r* = .335, *P* = .095
Relative power (W/kg)	*r* = .207, *P* = .310
Average speed (m/s)	*r* = .254, *P* = .210
Take-off speed (m/s)	*r* = .256, *P* = .207
Impulse (kg m/s)	*r* = .460, *P* = .018^*^
Flying time (ms)	*r* = .253, *P* = .212

*P < 0.05, **P < 0.01.

**Figure 3.**
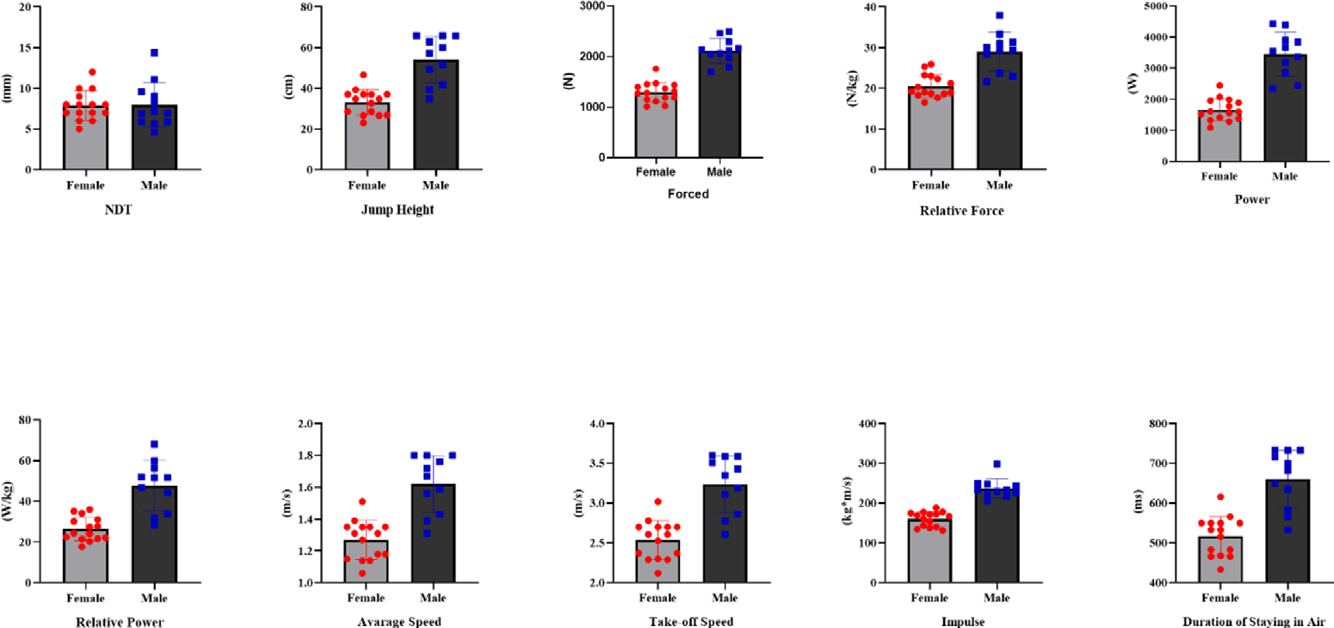
Comparison of NDT and CMJ parameters according to gender. CMJ = counter movement jump, NDT = navicular drop test.

Table 3 shows the *t* test results between the participants’ NDT results and CMJ parameters. According to this, there was no significant difference between the NDT results of the participants according to gender (*t* = 0.001, *d* = 0.006, *P* = .988). Among the CMJ parameters, jump height (*t* = 5.98, *d* = 2.375, *P* < .001), force (*t* = 3.77, *d* = 1.498, *P* < .001), relative force (*t* = 5.65, *d* = 2.24, *P* < .001), power (*t* = 8.57, *d* = 3.40, *P* < .001), relative power (*t* = 5.89, *d* = 2.341, *P* < .001), average speed (*t* = 5.97, *d* = 2.370, *P* < .001), take-off speed (*t* = 5.97, *d* = .2.371, *P* < .001), impulse (*t* = 9.21, *d* = 3.657, *P* < .001), flying time (*t* = 5.97, *d* = 2.373, *P* < .001) were higher in favor of men (Fig. 3).

**Table 3:** Comparison of NDT and CMJ parameters of participants according to gender.

Parameters	Gender	Mean ± SD	*t*	*d*	*P* value	95% CI	
						Lower	Upper
NDT (cm)	Male	7.94 ± 2.74	0.01	0.006	.988	−0.75	0.76
	Female	7.93 ± .1.96					
JH (cm)	Male	54.05 ± 11.44	5.98	2.375	<.001	1.33	3.38
	Female	33.13 ± 6.28					
Force (N)	Male	1940.76 ± 623.87	3.77	1.498	<.001	0.60	2.37
	Female	1297.46 ± 195.79					
RF (N/kg)	Male	28.99 ± 4.78	5.65	2.244	<.001	1.22	3.23
	Female	20.54 ± 2.82					
Power (W)	Male	3450.57 ± 700.99	8.57	3.404	<.001	2.15	4.62
	Female	1658.63 ± 359.72					
RP (W/kg)	Male	47.67 ± 12.34	5.89	2.341	<.001	1.30	3.34
	Female	26.34 ± 5.78					
AS (m/s)	Male	1.62 ± 0.17	5.97	2.370	<.001	1.33	3.38
	Female	1.26 ± 0.12					
TS (m/s)	Male	3.23 ± 0.35	5.97	2.371	<.001	1.33	3.38
	Female	2.53 ± 0.24					
Impulse (kg m/s)	Male	237.30 ± 24.19	9.21	3.657	<.001	2.35	4.93
	Female	160.34 ± 18.46					
FT (ms)	Male	660.36 ± 72.72	5.97	2.373	<.001	1.33	3.38
	Female	517.53 ± 49.30					

AS = average speed, FT = flying time, JH = hump height, NDT = navicular drop test, RF = relative force, RP = relative power, TS = take-off speed.

**Figure 4.**
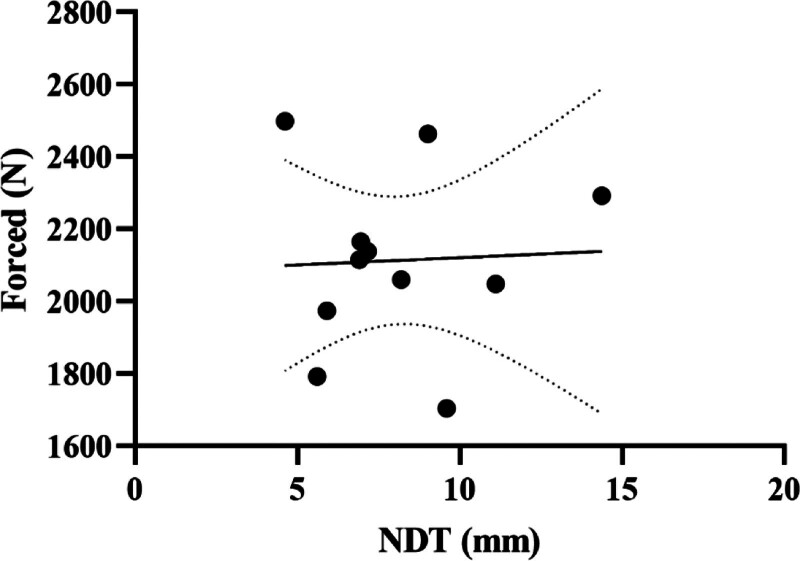
Regression analysis results between NDT and forced strength of male volleyball players: NDT = navicular drop test.

Table 4 shows the results of the linear regression analysis between the participants’ NDT results and CMJ performance parameters of male volleyball players. According to this, the participants’ force parameter had a positive relationship with NDT (*t* = 12.93, *P* = .049) (Fig. 4). There was no relationship between NDT and jump height, relative force, power, relative power, average speed, take-off time, impulse, and flying time [*F*_(1, 9)_ = 90.48, *P* = .081].

**Table 4:** Results of linear regression analysis between NDT results and CMJ performance parameters of male volleyball players.

Parameters	Estimate	SE	*t*	*P* value		*F*
					*R * ^2^	*P* value
Jump height (cm)	−8.69	17.31	−0.63	.639	0.998	90.48 .081
Force (N)	0.04	0.45	12.93	.049^*^		
Relative force (N/kg)	−12.57	63.40	2.93	.209		
Power (W)	−0.01	0.04	−1.70	.338		
Relative power (W/kg)	6.33	19.89	−2.40	.251		
Average speed (m/s)	−741.78	776.59	−10.82	.059		
Take-off speed (m/s)	−235.75	412.37	1.08	.473		
Impulse (kg m/s)	−0.21	3.84	0.94	.517		
Flying time (ms)	4.08	3.31	4.44	.141		

*P < 0.05.

**Figure 5.**
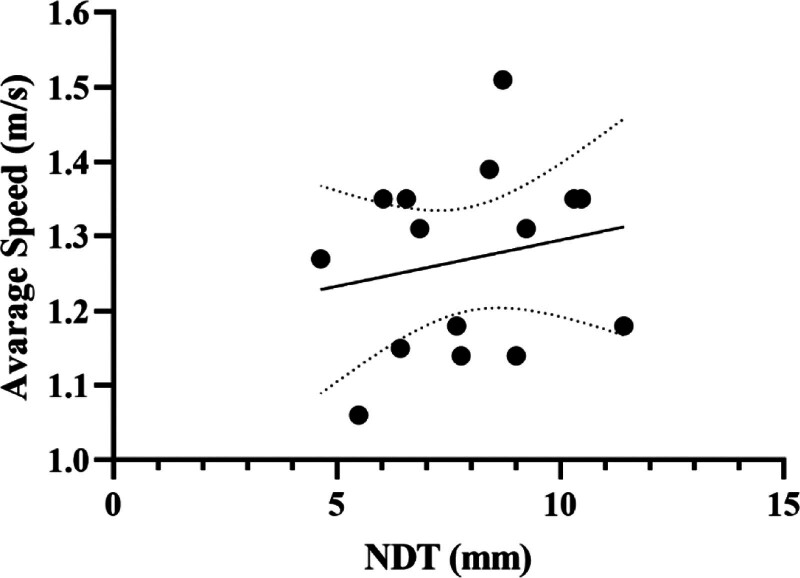
Regression analysis results between NDT and average speed of male volleyball players: NDT = navicular drop test.

Table 5 shows the results of linear regression analyses between NDT results and CMJ parameters of female volleyball players. Accordingly, a significant negative relationship was found between the average speed of the participants and NDT (*t* = −3.52, *P* = .017) (Fig. 5). There was no significant interaction between jump height, force, relative force, power, relative power, take-off speed, impulse, and flying time [*F*_(5, 9)_ = 1.88, *P* = .252].

**Table 5:** Results of linear regression analysis between NDT results and CMJ performance parameters of female volleyball players.

Parameters	Estimate	SE	*t*	*P* value	*R * ^2^	*F*
						*P* value
Jump height (cm)	7.34	4.88	1.50	.193	0.772	1.883 .252
Force (N)	−0.03	0.04	−0.62	.560		
Relative force (N/kg)	21.31	9.70	2.19	.080		
Power (W)	−0.004	0.002	−2.23	.076		
Relative power (W/kg)	−14.63	7.26	−2.01	.100		
Average speed (m/s)	−780.52	221.40	−3.52	.017^*^		
Take-off speed (m/s)	−254.95	232.56	−1.09	.323		
Impulse (kg m/s)	0.18	0.39	0.46	.665		
Flying time (ms)	2.89	1.43	2.01	.100		

*P < 0.05.

## 4. Discussion

This study aimed to explore the relationship between pes planus (flatfoot) levels and various parameters of CMJ performance among amateur male and female volleyball players. Our findings revealed distinct gender differences in CMJ parameters, with force in males and average speed in females showing significant associations with NDT results. These results underscore the importance of considering pes planus levels in sports performance evaluations and training programs as mentioned in other studies,^[30,31]^ particularly for sports requiring jumping abilities such as volleyball.

Contrary to our hypothesis (H1a) that CMJ parameters would be broadly affected by NDT results, significant associations were limited to specific parameters (force for males and average speed for females). This suggests that the impact of pes planus on jump performance may be more nuanced and variable across genders than previously understood.^[32–34]^ Furthermore, the lack of significant difference in NDT scores between genders, yet significant variations in all CMJ measures, highlights the complex interplay of foot structure with other biomechanical and physiological factors influencing jump performance.

Our findings align with studies suggesting that physical attributes such as height, weight, and leg length significantly correlate with CMJ performance, reflecting the multifaceted nature of athletic performance.^[35,36]^ However, the specific impact of pes planus on these relationships remains underexplored in the literature, highlighting a gap our study begins to address.

Akalan et al^[33]^ investigated the effects of pes planus on lower extremity kinematics and jumping performance during vertical jump. Performance and kinematics compared between volleyball players with flexible pes planus and normal foot. They found that vertical jump height was significantly lower in flatfoot players than in controls. They also found that peak pelvic tilting and hip flexion were reduced, although peak ankle dorsiflexion significantly increased during the jumping and landing phases.^[33]^ The research conducted by Akalan et al^[33]^ and a subsequent study examining the impact of pes planus on volleyball players’ performance share a focus on the implications of flatfoot on athletic abilities but diverge in methodology and findings. This study and our study recognize pes planus as influencing performance; however, Akalan et al^[33]^ spotlight a decrease in vertical jump height among flatfooted players, while the latter study identified specific CMJ parameters, such as force and average speed as significantly affected by pes planus. Akalan et al^[33]^ emphasized biomechanical alterations during jump phases, whereas the present study provides a broader analysis of CMJ metrics and their correlation with physical attributes, underlining the complex interplay between pes planus and volleyball performance, with significant gender disparities in CMJ outcomes and a nuanced understanding of the impact of flatfoot.

Şahin et al^[34]^ investigated the effects of pes planus deformity on the balance and vertical jump performance of athletes. Fifty athletes were included in the study, and the presence of pes planus was assessed using the Feiss line. In Addition, the participants’ balance and jump performance were recorded. The study reported that athletes with pes planus exhibited adversely affected balance and vertical jump performances.^[34]^ Şahin et al^[34]^ and our study investigated the ramifications of pes planus on athlete performance, yet they employed distinct methodologies and focused on varying performance outcomes. Our study utilized the NDT to assess pes planus and examine a broad spectrum of CMJ performance parameters among volleyball players, identifying gender-specific impacts and a strong association between pes planus and certain CMJ metrics. Conversely, Şahin et al^[34]^ applied the Feiss line for pes planus evaluation and concentrated on the general effects on balance and vertical jump performance, without explicitly focusing on gender differences or a wide range of jump performance indicators. While both investigations underscore the negative influence of pes planus on athletic abilities, the present study provides a more granular analysis of how pes planus levels correlate with specific CMJ parameters and physical characteristics, highlighting the complexity of their impact on performance.

Dikici and Demirdel^[37]^ conducted research to understand how the severity of pes planus (flatfoot) affects lower extremity performance in young adults with this condition. The study involved 53 young adults with asymptomatic flexible pes planus, utilizing the NDT to assess flatfoot severity and employing balance and jump tests to evaluate functional performance. The results indicated a weak negative correlation between pes planus severity and balance performance, particularly in anterior and posteromedial directions, without affecting vertical jump capabilities.^[37]^ Our study and that of Dikici and Demirdel^[37]^ both examine the implications of pes planus on physical performance, utilizing the NDT for evaluation. While we explored its effect on CMJ parameters in volleyball players, identifying significant gender-specific associations, Dikici and Demirdel^[37]^ focused on young adults with flexible pes planus, finding a weak negative correlation between pes planus severity and balance, but no impact on vertical jump. These findings illuminate the complex influence of pes planus across different athletic and demographic contexts, underscoring the condition’s varied effects on balance and dynamic sports performance.

Tudor et al^[38]^ investigated the potential relationship between foot arch flatness and an array of motor skills essential for athletic performance. The research involved scanning the feet of 218 children, aged between 11 and 15 years, to ascertain the arch index. This index was adjusted for age-related variations before the cohort was stratified into 4 categories based on the degree of foot flatness. The analysis sought to identify any significant correlations between foot arch height and 17 distinct motor abilities. However, the findings indicated no significant association between arch height and athletic performance, as evidenced by the lack of significant correlations across the evaluated motor skills. Furthermore, the division of the sample into quartiles based on foot arch flatness failed to elucidate any differences in athletic capabilities among the groups.^[38]^ Our study and that of Tudor et al^[38]^ examined the impact of foot morphology on athletic performance, albeit with divergent outcomes. We focused on how pes planus affects CMJ performance in volleyball players, finding significant correlations between pes planus levels and specific CMJ metrics. However Tudor et al,^[38]^ did not observe a significant relationship between foot arch flatness and a broad array of motor skills in children.

Marotta et al,^[39]^ aimed to assess the influence of proprioceptive mat training on plantar pressure and athletic performance in semiprofessional volleyball players. Employing a quasi-experimental design, nineteen semiprofessional volleyball players were divided into 2 groups: an experimental group that underwent specific proprioceptive and balance training on mats, and a control group that participated in a nonspecific sham protocol. Plantar pressure was measured using a baropodometric platform, and jump performance, including countermovement and squat jumps, was evaluated through an inertial measurement unit. The experimental group demonstrated a statistically significant improvement in plantar load distribution and a reduction in hindfoot pressure, compared to the control group. Additionally, the experimental group exhibited enhanced peak landing forces and increased concentric power output.^[39]^ Our study and that of Marotta et al^[39]^ addressed factors affecting jump performance in volleyball players, albeit through different lenses. We explore the relationship between pes planus levels and CMJ performance, identifying significant correlations with specific CMJ metrics. Marotta et al^[39]^ focused on the impact of proprioceptive mat training on plantar pressures and jump performance, demonstrating enhancements in load distribution and jump dynamics. While our research highlights the influence of anatomical characteristics on performance, Marotta et al^[39]^ provided evidence for the effectiveness of targeted training interventions, showcasing the interplay between physical attributes and performance-enhancing strategies in volleyball athletes.

The examination of the correlation between age at sport and several physical and performance metrics in volleyball players highlights the complex impact of sustained sport engagement on the development of athletes. Anthropometric parameters like height, weight, leg length, foot breadth, and foot length have significant positive associations with sport age, indicating a unique pattern of growth and development impacted by ongoing volleyball training.^[40]^ These modifications can be linked to volleyball’s energetic, repeated motions, which can encourage particular adaptations in the structure and composition of the body.^[41]^ In a similar vein, the correlation between increased strength and impulse producing capacity and athletic age suggests that prolonged training may play a part in fostering the neuromuscular efficiency and technical skills necessary for volleyball performance.^[42,43]^ On the other hand, the lack of a significant correlation with parameters like jump height, NDT, leg length at 90° flexion, and different power and speed measurements may suggest that these traits are more reliant on an athlete’s unique physiological makeup or reach a plateau early in their career.^[44]^ This disparity highlights the need for a tailored training program that emphasizes both the maintenance and development of traits that seem to be less impacted by the amount of volleyball played, in addition to the development of physical attributes that are positively correlated with sporting age.

The limitations of our study include the relatively small sample size and the focus on amateur athletes, which may limit the generalizability of our findings to professional or elite populations. Additionally, the cross-sectional design precludes causal inferences between pes planus levels and CMJ performance.

## 5. Conclusion

Our study provides valuable insights into the influence of pes planus on jump performance among amateur male and female volleyball players. The findings suggest that the pes planus level has a gender-specific impact on certain aspects of CMJ performance, with force in males and average speed in females being notably affected. These results emphasize the importance of considering individual biomechanical characteristics, such as foot structure, in the assessment and training of athletes, particularly in sports where jumping is a critical skill. Despite the limitations related to the sample size and the study’s cross-sectional nature, our research highlights the need for further exploration into the complex interactions between foot morphology and athletic performance. Future studies should aim to elucidate the mechanisms underlying these relationships and explore targeted interventions to optimize performance and reduce the risk of injury in athletes with pes planus. The study’s conclusions shed light on the complex relationship that exists between a volleyball player’s sporting age and the evolution of important performance and physical characteristics. Overall, this study shows how important it is for volleyball players to engage in sports and train continuously to improve physically. It also emphasizes the need for individualized training methods to target performance attributes that are less affected. Coaches, athletes, and sport scientists who want to maximize training plans for volleyball players at different phases of their athletic careers may find this material to be quite helpful.

## Acknowledgments

The authors express their gratitude to Princess Nourah bint Abdulrahman University Researchers Supporting Project number (PNURSP2024R535), Princess Nourah bint Abdulrahman University, Riyadh, Saudi Arabia for funding this research. In addition, authors would like to thank participants for their trust and help to make this study possible.

## Author contributions

**Conceptualization:** Ahmet Kurtoğlu, Bekir Çar, Safaa Mostafa Elkholi.

**Data curation:** Ahmet Kurtoğlu, Özgür Eken, Gözde Demir.

**Formal analysis:** Ahmet Kurtoğlu, Özgür Eken, Musa Türkmen.

**Funding acquisition:** Ahmet Kurtoğlu, Madawi H. Alotaibi, Safaa Mostafa Elkholi.

**Investigation:** Ahmet Kurtoğlu, Bekir Çar, Edi Setiawan.

**Resources:** Ahmet Kurtoğlu, Özgür Eken, Edi Setiawan.

**Software:** Ahmet Kurtoğlu.

**Supervision:** Ahmet Kurtoğlu, Bekir Çar, Safaa Mostafa Elkholi.

**Writing – original draft:** Ahmet Kurtoğlu, Bekir Çar, Özgür Eken, Gözde Demir, Musa Türkmen, Edi Setiawan, Madawi H. Alotaibi, Safaa Mostafa Elkholi.

**Writing – review & editing:** Ahmet Kurtoğlu, Bekir Çar, Özgür Eken, Gözde Demir, Musa Türkmen, Edi Setiawan, Madawi H. Alotaibi, Safaa Mostafa Elkholi.

**Methodology:** Ahmet Kurtoğlu, Bekir Çar, Özgür Eken, Gözde Demir.

**Visualization:** Bekir Çar, Özgür Eken, Gözde Demir, Musa Türkmen.

**Project administration:** Safaa Mostafa Elkholi.

**Validation:** Ahmet Kurtoğlu, Bekir Çar, Safaa Mostafa Elkholi.
